# A novel splicing mutation identified in a DMD patient: a case report

**DOI:** 10.3389/fped.2023.1261318

**Published:** 2023-11-20

**Authors:** Yuting Wen, Luo Yang, Gan Shen, Siyu Dai, Jing Wang, Xiang Wang

**Affiliations:** ^1^ Department of Obstetrics and Gynecology, Key Laboratory of Obstetric, Gynecologic and Pediatric Diseases and Birth Defects of Ministry of Education, West China Second University Hospital, Sichuan University, Chengdu, China; ^2^ Department of Urology & Pelvic Surgery, West China School of Public Health and West China Fourth Hospital, Sichuan University, Chengdu, China; ^3^ Department of Obstetrics and Gynecology, West China Second University Hospital of Sichuan University, Chengdu, China; ^4^ Key Laboratory of Birth Defects and Related Diseases of Women and Children, Ministry of Education, Sichuan University, Chengdu, China; ^5^ NHC Key Laboratory of Chronobiology, Sichuan University, Chengdu, China

**Keywords:** DMD, Duchenne muscular dystrophy, preimplantation genetic diagnosis, splicing mutation, case report

## Abstract

**Background:**

Duchenne muscular dystrophy (DMD, ORPHA:98896) is a lethal X-linked recessive disease that manifests as progressive muscular weakness and wasting. Mutations in the dystrophy gene (*DMD*) are the main cause of Duchenne muscular dystrophy.

**Case presentation:**

This study aims to determine novel mutations of DMD and help preimplantation genetic diagnosis (PGD) for family planning. Here present a 4-year-old Chinses boy with DMD, whole-exome sequencing (WES) was performed to identify the molecular basis of the disease. It was confirmed that the boy carried a novel hemizygous mutation of NC_000023.11(NM_004006.3): c.5912_5922 + 19delinsATGTATG in DMD which inherited from his mother. This led to the aberrant splicing of DMD which demonstrated by a minigene splicing assay and further resulted in the impairment of the dystrophy protein.

**Conclusions:**

Our study discovered a novel splicing mutation of *DMD* in a DMD patient, which expands the variant spectrum of this gene and provide precise genetic diagnosis of DMD for timely therapy. Meanwhile, this finding will supply valuable information for preimplantation genetic diagnosis.

## Introduction

Duchenne muscular dystrophy (DMD) is a lethal pediatric muscular dystrophy, which mainly affects males with a prevalence of one in 5,000–6,000 (1, 2). It is an X-linked recessive disease, which manifests as progressive muscular weakness and wasting (2). The disease usually is commonly diagnosed in early childhood, around 3–5 years old, with symptoms such as delayed motor development, elevated levels of serum creatine kinase (CK), abnormal gait, poor running and climbing, and frequent falls. In some cases, language or learning problems have been observed (2, 3). As the affected individual ages, the disease progresses rapidly and can lead to the development of cardiac and respiratory complications (3, 4). Therefore, patients usually lose the ability to stand and run and have limited mobility before the age of 12. Patients with DMD will often die due to cardiomyopathy or respiratory failure. The median life expectancy of patients with DMD has significantly improved in recent decades (1, 5, 6). However, these patients still often died at a young age, primarily due to cardiomyopathy or respiratory failure. DMD poses a serious threat to the health of young adults and imposes a significant psychological and economic burden on families and society.

Genetically, DMD is caused by variants in the *DMD* gene. This gene is located at Xp21.2 and consists of 79 exons. At present, 34,890 variations of *DMD* have been reported in a *DMD* gene database (https://databases.lovd.nl/shared/genes/DMD). Approximately 60%–70% of mutations observed in DMD are deletions or duplications in single or multiple exons (7, 8). The remaining genetic changes are mainly related to small mutations, which include nonsense, missense, splice site variations, or small rearrangements like insertion/deletion, and small inversions (9–12). Splicing mutations, which can lead to disruption of existing splicing regulatory sequences, alterations in the open reading frame and inappropriate intron removal during splicing process, and ultimately the production of a defective protein (13), constitute approximately 3% of total mutations of *DMD*, and has been thought to an important causes for undiagnosed DMD (11, 14).

Preimplantation genetic diagnosis (PGD) is a clinically feasible technology for testing genetic defects of embryos fertilized *in vitro* to achieve a pregnancy without genetic disease. It has been developed mainly for couples carrying severe recessive or X-linked Mendelian disorders, structural chromosome abnormalities or mitochondrial disorders (15), like Klinefelter syndrome, Cystic fibrosis and DMD (16–18). In addition, it has been reported that early and accurate genetic diagnosis of mutations in *DMD* could prevent the recurrence of DMD by PGD (19). Thus, it is crucial to identify new variants in *DMD* for PGD, as well as prenatal diagnosis, genetic counseling, and gene therapy for DMD.

Herein, a novel mutation of c.5912_5922 + 19delinsATGTATG in *DMD* was identified in a 4-year-old boy through whole-exome sequencing (WES). Furthermore, this mutation resulted in abnormal splicing of the *DMD* transcripts due to the retention of 12 bp of intron 41 to the transcripts. Therefore, we deduced that this mutation ultimately gave rise to dysfunction of the dystrophy protein and further contributed to the development of DMD in this patient. Our study provided a precise genetic diagnosis for the patient and offered valuable assistance to his family in ensuring a healthy pregnancy through PGD.

## Case presentation

A 4-year-old boy being speculated as having DMD was investigated in this study. The patient is the first child of healthy parents, and there was no heredity and medical history within his family. The patient's parents were eager to understand the underlying genetic factors behind the patient's symptoms and be assisted in future pregnancy to prevent the recurrence of the same mutation in the new offspring by PGD. The patient complained of reeling gait, delay in motor development, Gowers' sign on rising from floor, and having difficulties climbing stairs, which are suggestive of DMD clinical phenotypes (2, 3).

**Figure 1:**
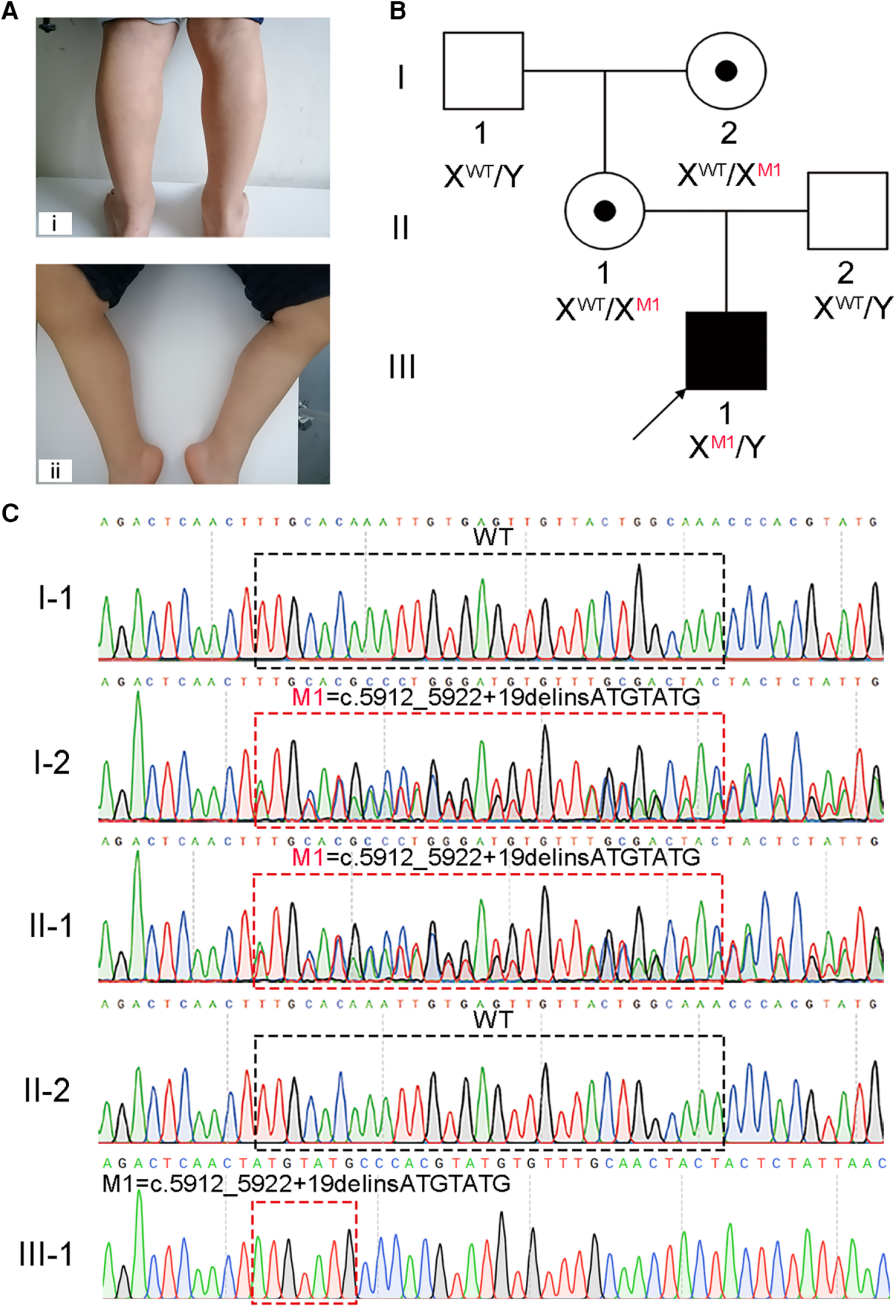
A hemizygous splicing mutation in the *DMD* gene identified in the boy with DMD. (**A**) Clinical signs of the patient. i and ii, muscle weakness and calf pseudohypertrophy. (**B**) Pedigree structure in the DMD family. The black square with black arrow indicates the patient. (**C**) Sanger sequencing verified the mutation of c.5912_5922 + 19delinsATGTATG in this family. The black and red dotted boxes respectively represent the mutation positions of the unaffected and affected members.

Upon general physical examination, fatigue, muscle weakness, and calf pseudohypertrophy were observed in the patient (Figure 1A). An electromyograph showed that the fasciculation potential could be seen at the resting state and appeared as a pathological interference phase when the medial femoris muscle contracted vigorously; low amplitude coupled with short-duration motor unit potentials (MUPs) was also found in the medial head of the gastrocnemius and the medial femoris muscle. The patient's nerve conduction velocity was found to be normal. These findings reflect myogenic injury in the patient. Interestingly, muscle power assessment, as determined by the Medical Research Council's scale (MRC scale) of muscle power, showed a slight reduction in muscle tone.

To confirm the clinical suspect of DMD, laboratory examination was performed on blood samples, and the results are presented in Supplementary Table 1. The patient's serum CK levels were significantly elevated to 11,614.7 U/L, which is 68 times the normal range. Other indicators, including creatine kinase MB (CK-MB), alanine transaminase (ALT), aspartate aminotransferase (AST), and lactate dehydrogenase (LDH) levels, were increased as well. The patient's family members had no abnormalities in biochemical analyses. Along with damage to muscle fibers, CK levels can also reflect myocardial damage. Moreover, cardiomyopathy is also observed in DMD patients and poses a severe threat to their life (4). Electrocardiograms and echocardiographs were performed on the patient, but no abnormalities were detected. Furthermore, after informed consent was obtained from the patient's parents, a gastrocnemius muscle biopsy was performed, which revealed that dystrophin protein production was severely reduced. The combined clinical presentation, physical examination, laboratory evidence, and imaging findings provide a diagnosis of DMD for this 4-year-old boy.

Molecular diagnosis is a precise approach to discover the genetic factors causing DMD and is helpful for family counseling and future gene therapy for DMD (20). We recommended to the patient's parents the multiplex ligation-dependent probe amplification (MLPA) analysis, the most reliable test for diagnosing DMD, and WES. Considering WES identifies a broader range and types of variants compared to MLPA, the patient's parents opted for WES. Thus, WES was carried out on the patient, and a hemizygous splicing mutation [NC_000023.11(NM_004006.3): c.5912_5922 + 19delinsATGTATG] of X-linked *DMD* was identified. This mutation was confirmed to be not previously annotated by searching common databases, such as the ExAC Browser, gnomAD, or the 1,000 genome Project. To further confirm the putative contribution of this mutation to the patient's presentation, Sanger sequencing was performed on the patient and his healthy family members. The results verified that the hemizygous mutation was inherited from his mother, which was passed on from his grandmother, while his father and grandfather did not carry this mutation (Figures 1B,C).

**Figure 2:**
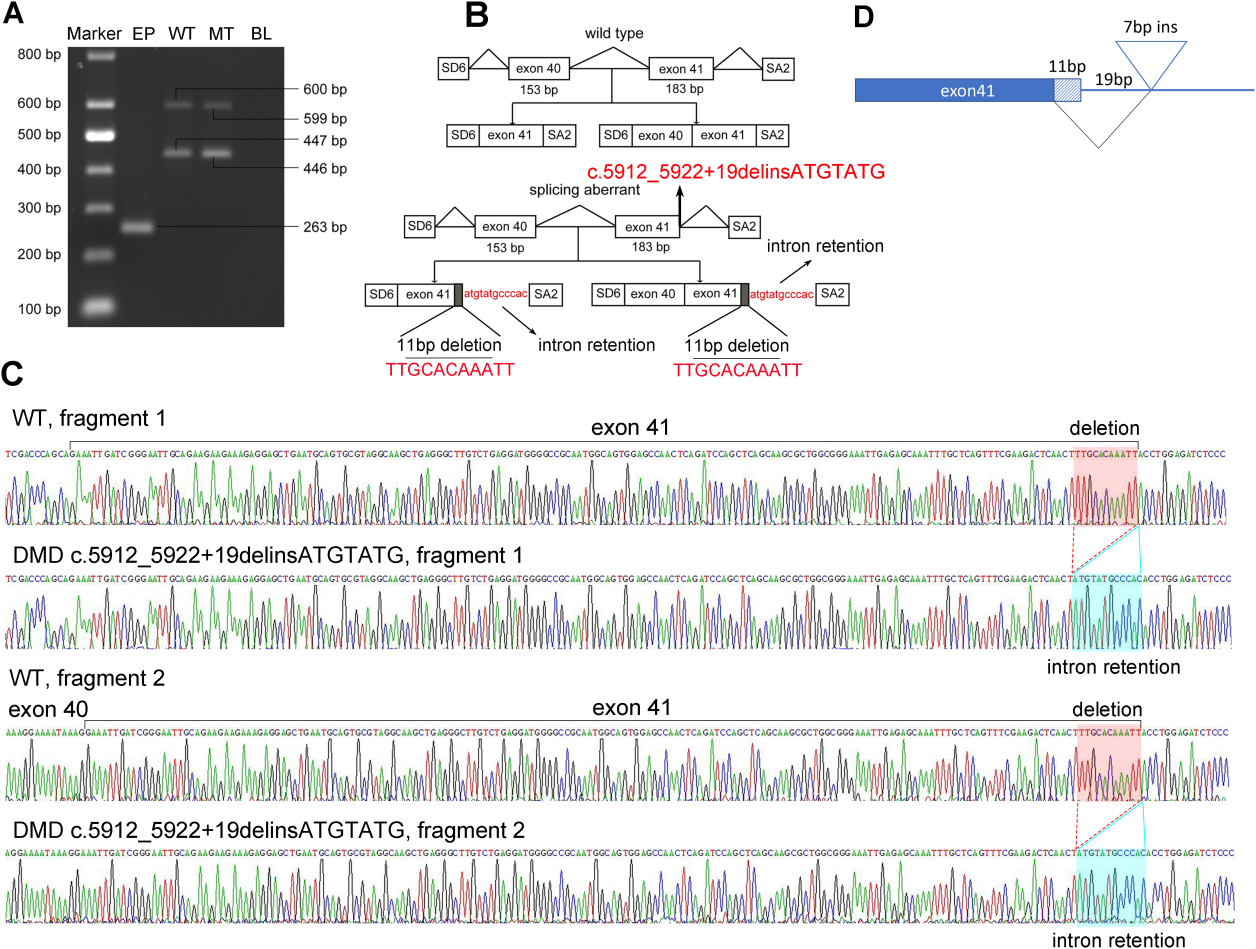
Pathogenic analysis of the hemizygous splicing mutation in *DMD* by minigene assay. (**A**) Electrophoretogram of RT-PCR products. Lane 1, marker; 2, the empty plasmid (EP) including SD6 and SA2; 3, the mutant plasmid (MT), 4, the wild-type plasmid (WT); 5, blank control (BL). (**B**) Schematic of the splicing models in this *DMD* mutation. (**C**) Sequencing result of the mutated cDNA showing the aberrant splicing of intron 41. (**D**) Schematic view of the splicing events in this *DMD* mutation.

To further understand the effect of the splicing mutation, a minigene assay was performed. The wild-type (WT) minigene plasmid consists of exon 40 (153 bp), intron 40 (851 bp), exon 41 (183 bp), and partial regions of intron 41 (167 bp) to construct a full length of 1,354 bp. After transfection into COS7 cells for 48 h, the transcripts were analyzed by RT-PCR. Gel electrophoresis revealed that the WT plasmid produced two fragments, indicating two splicing events (Figure 2A). The 446 bp fragment corresponded to exon 41 and the 599 bp fragment corresponded to exon 40 and exon 41, as confirmed by Sanger Sequencing (Figures 2B,C). Cells transfected with the mutant c.5912_5922 + 19delinsATGTATG plasmids also produced two fragments, each nearly equivalent to the WT (Figure 2A). However, subsequent Sanger sequencing revealed that the two mutant fragments exhibited a deletion of 11 bp (TTGCACAAATT) in exon 41 and a retention of 12 bp (ATGTATGCCCAC) in intron 41 (Figures 2B,C). Taken together, these findings suggest that the mutation in the *DMD* gene produced an aberrantly spliced cDNA (Figure 2D), which accounts for DMD presentation.

## Discussion and conclusions

In this study, a 4-year-old boy with DMD was identified, and genetic characterization revealed a mutation of c.5912_5922 + 19delinsATGTATG in the *DMD* gene. This mutation is a rare splicing mutation, which has not been previously reported, and affects the normal splicing of RNA through deleting and inserting some bases and causes the mRNA to be translated out of frame. This subsequently results in the abnormal translation of dystrophin and thus leads to a negative impact on its expression, as indicated by the muscle biopsy.

Dystrophin is a 427-kDa cytoskeletal protein encoded by the *DMD* gene containing four main functional domains (10, 21): an actin-binding amino-terminal domain (ABD1), which plays an important role in connecting dystrophin with the subsarcolemmal actin network via directly binding to F-actin; a central rod domain, with its tryptophan residues in the spectrin-like repeats that bind membrane phospholipids *in vitro* (22); a cysteine-rich domain, which helps dystrophin attach to the glycoprotein complex on the sarcolemma (23); and a carboxyl-terminus, which links to the intracellular syntrophins (24). Dystrophin is an essential protein for muscle contraction and relaxation. In the dysfunction of dystrophin, myofibers become extremely vulnerable and susceptible to injury. Therefore, the mass and function of normal muscles will gradually be lost due to multiple degeneration and regeneration cycles of damaged muscles, which further leads to progressive muscle weakness and the development of DMD (25, 26).

DMD patients' symptoms are often accompanied by a series of complications. Interestingly, language competency and average intelligence quotient (IQ) of a third of DMD patients may be lower than those of the normal population (27, 28). Patients with DMD also have a higher incidence of neuropsychiatric disorders (29). These complications, which were present at birth but did not show progressive development with the patients' age, were associated with the dystrophin isoforms expressed in the human central nervous system, such as Dp140, Dp116 and Dp71. These isoforms are generated by the internal promoters of *DMD* using a unique first exon that splices into, 45, 56, and 63 (30). However, the mutation found in this study is located at Dp427m, which is predominant in muscles. Thus, the patient did not show these neurological deficit-related phenotypes. In addition, some patients may develop early respiratory failure (31) or have poor cardiopulmonary function (32, 33). In this patient, no respiratory and cardiac complications were found. This may be due to the patient's young age, since these complications were in the early stage of ongoing development and thus had no phenotype. Therefore, annual surveillance of the respiratory and cardiac function should be performed.

Currently, the major therapies for DMD are physiotherapy and glucocorticoids therapy. Although they can improve a patients' muscle function and delay the loss of ambulation, severe side effects, including growth suppression, obesity, behavioral disturbances, and others, prevent their prevalent application (2, 34, 35). Response to physiotherapy is different in each patient, and the type and dosage of glucocorticoids are difficult to select. Additionally, physiotherapy and glucocorticoid therapy can delay the occurrence of DMD but are not curative. The development of genetic therapy, such as antisense oligonucleotides and genome editing, has been beneficial to cure DMD (36–38). However, these treatments are only suitable for specific mutations within the population and are not universally applicable. Additionally, they should be administered before the dysfunction of dystrophin. Furthermore, it is not known if there are adverse consequences to these treatments, and these issues limit their clinical use. Overall, a genetic diagnosis of DMD as early as possible is of vital importance for early and appropriate treatment and a better outcome. In this case, the patient is 4 years old and has not yet presented a serious DMD phenotype. This allows managements including medical and physical treatments to have the potential to effectively delay the development of DMD. Meanwhile, PGD is a valid method to reduce the risk of reappear of DMD in affected families. The early and precise genetic diagnosis of DMD in our patient provides a valuable information for PGD, thus helps his parents deliver a healthy child and also effectively prevents the birth of DMD patients carrying the same mutation.

In conclusion, we have discovered a novel hemizygous mutation of *DMD* in a DMD patient, which expands the variant spectrum of this gene and further helps in PGD for family planning. Additionally, we deduced the underlying splicing effect of this mutation, which might help gene therapy and drug research for these kinds of variants.

## Data Availability

The original contributions presented in the study are included in the article/**Supplementary Material**, further inquiries can be directed to the corresponding authors.
