# Supplementary material for: A novel splicing mutation identified in a DMD patient: a case report
Source: Front Pediatr. 2023 Nov 20;11:1261318. doi: 10.3389/fped.2023.1261318 (PMC10694253; doi:10.3389/fped.2023.1261318)
Supplement: Supplementary file 1 [file Table1.docx]

**Table 1. Biochemical analyses for the patient show muscle-specific changes.**

| **Lab finding** | **Values** | **Normal ranges** |
| --- | --- | --- |
| CK (U/L) | 11614.7 | 30-170 |
| CK-MB (U/L) | 334.8 | 0-24 |
| ALT (U/L) | 438.7 | 0-50 |
| AST (U/L) | 314.1 | 0-55 |
| LDH (U/L) | 822.4 | 140-270 |

CK: creatine kinase; CK-MB: creatine kinase MB; ALT: alanine transaminase; AST: aspartate aminotransferase; LDH: lactate dehydrogenase.
